# Supplementary material for: Streptomyces pratensis S10 Promotes Wheat Plant Growth and Induces Resistance in Wheat Seedlings against Fusarium graminearum
Source: J Fungi (Basel). 2024 Aug 15;10(8):578. doi: 10.3390/jof10080578 (PMC11355099; doi:10.3390/jof10080578)
Supplement: Supplementary file 1 [file jof-10-00578-s001.zip › jof-3125469-supplementary.pdf]

Streptomyces pratensis S10 promotes wheat plant growth and induces resistance in wheat seedlings against _Fusarium graminearum_

Supplementary Information

Xiaoman Tian[1#], Lifang Hu[2#], Ruimin Jia[2], Shang Cao[2], Yan Sun[2], Xiaomin Dong[2], Yang Wang[2]*

[1]Yangling Vocation and Technical College, Yangling, Shaanxi 712100, P.R. China

[2]College of Plant Protection, Northwest A&F University, Yangling, Shaanxi 712100, P.R. China

[#]These authors contributed equally to this study

*Corresponding Author: wangyang2006@nwafu.edu.cn

Table S1 Primers used in this study for defense-related genes and fungal biomass determination

Name	Primer type	Sequence (5'-3')	Use
Ta54227	F	ATCCACGTCACCACTTTCAA	used as a reference for wheat
	R	TGCTTGGAGATCCACATTTG	
TaPR1.1	F	GAGAATGCAGACGCCCAAGC	qPCR
	R	CTGGAGCTTGCAGTCGTTGATC	
TaPR2	F	AGGATGTTGCTTCCATGTTTGCCG	qPCR
	R	AAGTAGATGCGCATGCCGTTGATG	
TaPAL1	F	CAGCACAGCAAAACCGTATCG	qPCR
	R	ACCTTGTCCACCTCTTCGCC	
TaPR3	F	AGAGATAAGCAAGGCCACGTC	qPCR
	R	GGTTGCTCACCAGGTCCTTC	
TaPR4a	F	CGAGGATCGTGGACCAGTG	qPCR
	R	GTCGACGAACTGGTAGTTGACG	
TaPR9	F	CAAGGTGAACTCGTGATGGA	qPCR
	R	TTGAGGATTCAACCGTCGTT	
TaPR5	F	ACAGCTACGCCAAGGACGAC	qPCR
	R	CGCGTCCTAATCTAAGGGCAG	
TaPDF1.2		TGAGGGAAGTCGATCTGAATGAG	qPCR
		GCCCAGTTCCACTGTTTTCACT	

FGSG_09530	F	AGGTTGAGGACCAGATGCG	qRT-PCR for determining the expression of tubulin
	R	CCTGGATAGAGGTGGAGTTTC	

Table S2 Genes involved in the modulation of plant hormones

Gene ID	Gene name	Product	Pathway
gene1387	-	1-aminocyclopropane-1-carboxylate deaminase	ACC catabolism
gene0978	*trpS*	tryptophanyl-tRNA synthetase	L-tryptophan production
gene2150	*prnA*	tryptophan halogenase	
gene2166	*TDO2*	tryptophan 2,3-dioxygenase	
gene5280	-	tryptophan 2-monooxygenase	
gene6113	*trpA*	tryptophan synthase alpha chain	
gene6114	*trpB*	tryptophan synthase beta chain	
gene6115	*trpC*	indole-3-glycerol phosphate synthase	
gene8191	*TDO2*	tryptophan 2,3-dioxygenase	
gene8192	*TDO2*	tryptophan 2,3-dioxygenase	
gene8325	*trpS*	tryptophanyl-tRNA synthetase	
gene1100	*mca*	mycothiol S-conjugate amidase	IAA production; IAM pathway
gene1981	*pncC*	nicotinamide-nucleotide amidase	
gene2611	-	N-acetylmuramoyl-L-alanine amidase	
gene2632	*srfJ*	glucosylceramidase	
gene2709	-	neuramidase	
gene1471	-	amidase	
gene2761	-	amidase	
gene2961	*amiE*	amidase	
gene4231	-	N-acetylmuramoyl-L-alanine amidase	
gene4590	*pncA*	amidase	
gene6459	*amiA*	N-acetylmuramoyl-L-alanine amidase	
gene7411	*pncA*	nicotinamidase/pyrazinamidase	
gene7554	-	N-acetylmuramoyl-L-alanine amidase	
gene7771	-	penicillin amidase	
gene8292	-	N-acetylmuramoyl-L-alanine amidase	
gene5280	-	tryptophan 2-monooxygenase	
gene6729	-	nitrilase	IAA production; IAN pathway
gene6582	*aofH*	monoamine oxidase	IAA production, TAM pathway
gene0920	*gabD*	succinate-semialdehyde dehydrogenase	
gene1050	-	aldehyde dehydrogenase	
gene1051	-	aldehyde dehydrogenase	
gene1869	*betB*	betaine-aldehyde dehydrogenase	
gene1893	-	aldehyde dehydrogenase	
gene2253	*xylC*	benzaldehyde dehydrogenase	
gene2385	*mhpF*	acetaldehyde dehydrogenase	
gene3213	*betB*	betaine-aldehyde dehydrogenase	
gene3341	-	aldehyde dehydrogenase	
gene4020	*aldB*	aldehyde dehydrogenase	
gene4021	*aldB*	aldehyde dehydrogenase	

gene4295	*gabD*	succinate-semialdehyde dehydrogenase	
gene4297	*gabD*	succinate-semialdehyde dehydrogenase	
gene4495	-	aldehyde dehydrogenase	
gene4766	*betB*	betaine-aldehyde dehydrogenase	
gene5283	-	aldehyde dehydrogenase	
gene5519	-	aldehyde dehydrogenase	
gene5656	-	aldehyde dehydrogenase	
gene5899	*aldH*	aldehyde dehydrogenase	
gene6911	*proA*	glutamate-5-semialdehyde dehydrogenase	
gene7000	*asd*	aspartate-semialdehyde dehydrogenase	
gene7080	*mmsA*	methylmalonate-semialdehyde dehydrogenase	
gene7250	*asd*	aspartate-semialdehyde dehydrogenase	
gene7870	-	aldehyde dehydrogenase	
gene7871	-	aldehyde dehydrogenase	
gene8036	-	aldehyde dehydrogenase	
gene8160	*asd*	aspartate-semialdehyde dehydrogenase	
gene2020	*miaA*	tRNA dimethylallyltransferase	CK biosynthesis and transformation
gene4299	*yagR*	xanthine dehydrogenase	

Table S3 Genes involved in iron transport and siderophore production

Gene ID	Gene name	Product	Pathway
gene4128	-	NADPH-dependent ferric siderophore reductase	Ferric iron reduction
gene5531	-	NADPH-dependent ferric siderophore reductase	
gene7198	-	NADPH-dependent ferric siderophore reductase	
gene7837	-	NADPH-dependent ferric siderophore reductase	
gene2035	-	siderophore biosynthesis protein IucA	Siderophore production
gene2036	-	siderophore biosynthesis protein IucA	
gene7202	-	siderophore biosynthesis protein IucA	
gene7203	-	siderophore biosynthesis protein IucA	
gene0976	*rpoE*	siderophore-interacting protein	
gene4677	*rpoE*	siderophore-interacting protein	
gene5041	*rpoE*	siderophore-interacting protein	
gene3953	*cysK*	putative siderophore biosynthesis protein SbnA	
gene1645	-	TesB-like acyl-CoA thioesterase 3	Enterobactin production
gene2099	*entH*	thioesterase	
gene2869	*entH*	thioesterase	
gene4889	*entH*	thioesterase	
gene4958	*tesB*	acyl-CoA thioesterase	
gene6065	*entH*	thioesterase	
gene6634	*entH*	thioesterase	
gene7071	*entH*	thioesterase	
gene7181	*tesB*	acyl-CoA thioesterase II	
gene2155	*entE*	2,3-dihydroxybenzoate-AMP ligase	
gene3537	*entE*	2,3-dihydroxybenzoate-AMP ligase	
gene5680	*entE*	2,3-dihydroxybenzoate-AMP ligase	
gene2131	*entB*	isochorismatase	
gene7952	*entB*	isochorismatase	
gene1122		iron transporter	Iron transport
gene2030	-	iron transporter	
gene2031	-	iron transporter	
gene2032	-	iron transporter	
gene0533	-	iron-siderophore ABC transporter substrate-binding protein	
gene0535	-	iron-enterobactin transporter ATP-binding protein	
gene0536	-	iron ABC transporter	
gene0537	-	iron ABC transporter permease	
gene0988	*sdhB*	succinate dehydrogenase iron-sulfur subunit	
gene1608	-	iron ABC transporter solute-binding protein	
gene1727	-	iron ABC transporter permease	
gene1728		iron-siderophore uptake system protein	
gene1729	-	iron dicitrate ABC transporter ATP-binding protein	

gene1859	*thiP*	iron ABC transporter permease
gene1860	*thiQ*	iron transporter ATP-binding protein
gene2738	-	iron ABC transporter permease
gene2740	-	iron dicitrate ABC transporter ATP-binding protein
gene3567	-	iron ABC transporter substrate-binding protein
gene3568	-	iron ABC transporter substrate-binding protein
gene3569	-	iron transporter
gene3570	-	iron transporter
gene3571	-	iron transporter
gene3572	-	iron transporter ATP-binding protein
gene4384	-	iron ABC transporter permease
gene4385	-	iron ABC transporter permease
gene5138	-	iron-sulfur protein
gene5774	-	iron ABC transporter permease
gene5792	-	iron transporter ATP-binding protein
gene5970	*iscU*	iron-sulfur cluster assembly scaffold protein
gene6274	-	iron-sulfur cluster insertion protein ErpA

Table S4 Genes involved in phosphate solubilization and transport

Gene ID	Gene name	Product	Pathway
gene7639	*ppx-gppA*	exopolyphosphatase	Degradation of inorganic polyphosphates
gene7965	*ppx-gppA*	exopolyphosphatase	
gene0634	*ppx-gppA*	exopolyphosphatase	
gene8025	*ppa*	Inorganic pyrophosphatase	
gene6442	*gpmB*	bifunctional RNase H/acid phosphatase	Organic phosphate solubilization
gene1189	*ppgK*	polyphosphate glucokinase	
gene1578	*pta*	phosphate acetyltransferase	
gene0914	*glpA*	glycerol-3-phosphate dehydrogenase	
gene3036	*gpr*	glyceraldehyde 3-phosphate reductase	
gene5088	*bacA*	UDP pyrophosphate phosphatase	
Gene6863	*phoH*	phosphate starvation-inducible protein PhoH and related proteins	
gene6414	*phoD*	alkaline phosphatase	
gene5687	*phoD*	alkaline phosphatase	
gene5056	*phoD*	alkaline phosphatase	
gene7367	*agaR*	alkaline phosphatase	
gene5954	-	alkaline phosphatase	
gene6499	-	alkaline phosphatase	
gene6500	-	alkaline phosphatase	
gene2083	-	alkaline phosphatase family protein	
gene3114	-	alkaline phosphatase family protein	
gene0327	*pstB*	phosphate transport system ATP-binding protein	Phosphate transport
gene0328	*pstA*	phosphate transport system permease protein	
gene0329	*pstC*	phosphate transport system permease protein	
gene0331	*pstS*	phosphate transport system substrate-binding protein	
gene0422	*phoU*	phosphate transport system protein	
gene0326	-	phosphate transporter regulator	
gene0326	-	inorganic phosphate transporter, PiT family	
gene5854	-	inorganic phosphate transporter, PiT family	
gene5854	-	carboxyvinyl-carboxyphosphonate phosphorylmutase	Degradation of phosphonates
gene5988	*pgl*	6-phosphogluconolactonase	D-gluconate production
gene4562	-	6-phosphogluconate dehydrogenase	
gene0354	-	6-phosphogluconate dehydrogenase	
gene8467	*gnd*	6-phosphogluconate dehydrogenase	
gene5988	*gnl*	gluconolactonase	
gene2258	*gntT*	gluconate permease	
gene2259	*gntT*	gluconate permease	
gene5617	*gntK*	gluconate kinase	
gene5618	-	gluconate transporter	
gene0405	*mshA*	D-inositol-3-phosphate glycosyltransferase	Other genes

gene0427	*ispD*	2-C-methyl-D-erythritol 4-phosphate cytidylyltransferase	involved in phosphate solubilization and transport
gene0428	*ispF*	2-C-methyl-D-erythritol 2,4-cyclodiphosphate synthase	
gene0466	*lacC*	tagatose 6-phosphate kinase	
gene0468	*nagA*	N-acetylglucosamine-6-phosphate deacetylase	
gene0473	*otsB*	trehalose 6-phosphate phosphatase	
gene0474	*otsA*	trehalose 6-phosphate synthase	
gene0500	*murQ*	N-acetylmuramic acid 6-phosphate etherase	
gene0646	*pdxH*	pyridoxamine 5'-phosphate oxidase	
gene0647	*pdxH*	pyridoxamine 5'-phosphate oxidase	
gene0681	*pdxH*	pyridoxamine 5'-phosphate oxidase	
gene1189	*ppgK*	polyphosphate glucokinase	
gene1579	*pfk*	ATP-dependent phosphofructokinase	
gene1790	*gpsA*	glycerol-3-phosphate dehydrogenase (NAD(P)+)	
gene5349	*pdxT*	5'-phosphate synthase pdxT subunit	
gene5350	*pdxS*	pyridoxal 5'-phosphate synthase pdxS subunit	
gene5355,5356	*pgsA*	CDP-diacylglycerol--glycerol-3-phosphate 3-phosphatidyltransferase	
gene5423	*plsC*	1-acyl-sn-glycerol-3-phosphate acyltransferase	
gene5439	*gapA*	glyceraldehyde 3-phosphate dehydrogenase	
gene7683	*prsA*	ribose-phosphate pyrophosphokinase	
gene8081	*hppA*	K (+)-stimulated pyrophosphate-energized sodium pump	
gene8196	*fbaA*	fructose-bisphosphate aldolase, class II	

Table S5 Genes involved in nitrogen fixation and nitrogen metabolism

Gene ID	Gene name	Product	Pathway
gene1827	*glnB*	nitrogen regulatory protein P-II 1	Nitrogen fixation
gene2988	*glnB*	nitrogen regulatory protein P-II 1	
gene0731	-	flavodoxin	
gene2266	-	flavodoxin	
gene7566	-	flavodoxin	
gene1657	*iscS*	cysteine desulfurase	
gene3550	*sufS*	cysteine desulfurase	
gene5971	*sufS*	cysteine desulfurase	
gene6253	-	cysteine desulfurase	
gene6268	*iscS*	cysteine desulfurase	
gene6269	*iscS*	cysteine desulfurase	
gene2241	-	nitrate reductase (NADH)	Dissimilatory nitrate reduction
gene2958	-	nitrate reductase (NADH)	
gene4768	-	nitrate reductase (NADH)	
gene4769	-	nitrate reductase (NADH)	
gene4770	-	nitrate reductase (NADH)	
gene5277	-	nitrate reductase (NADH)	
gene6827-6829	*nirB*	nitrite reductase (NADH) large subunit	
gene6830	*nirD*	nitrite reductase (NADH) small subunit	
gene6838	*narI*	nitrate reductase gamma subunit	
gene6607, 7746	*narK*	MFS transporter, NNP family, nitrate/nitrite transporter	
gene6870, 6871	*ncd2*	nitronate monooxygenase	
6813	*NIT-6*	nitrite reductase (NAD(P)H)	Assimilatory nitrate reduction
gene6814, 6815, 7784	*nasA*	assimilatory nitrate reductase catalytic subunit	
gene6826	*nasB*	nitrate reductase (NAD(P)H)	
gene6825	*nirA*	ferredoxin-nitrite reductase	
gene4628	*napA*	electron transfer subunit alpha	Denitrification
gene4629	*napB*	electron transfer subunit beta	
gene5235	*narG*	nitrate reductase	
gene7505,7506	-	glutamate dehydrogenase	Glutamate metabolism
gene3404	*gldA*	glutamate dehydrogenase (NAD(P)+)	
gene5921	*gldA*	glutamate dehydrogenase (NAD(P)+)	
gene1080	*gldA*	glutamate dehydrogenase (NAD(P)+)	
gene6102	*gltB*	glutamate synthase (NADPH/NADH) large chain	
gene5520	*glnA*	glutamine synthetase	
gene6309	*glnA*	glutamine synthetase	

gene6322	*glnA*	glutamine synthetase	
gene6366	*glnA*	glutamine synthetase	
gene2404	-	nitrate/nitrite transporter ATP-binding	Nitrate/nitrite transporter
